# Diffuse Ultrasonic Wave-Based Damage Detection of Railway Tracks Using PZT/FBG Hybrid Sensing System

**DOI:** 10.3390/s22072504

**Published:** 2022-03-24

**Authors:** Xiangtao Sun, Chuanrui Guo, Lei Yuan, Qingzhao Kong, Yiqing Ni

**Affiliations:** 1 Department of Disaster Mitigation for Structures, Tongji University, Shanghai 200092, China; sunxt@tongji.edu.cn (X.S.); qkong@tongji.edu.cn (Q.K.); 2 National Rail Transit Electrification and Automation Engineering Technology Research Center (Hong Kong Branch), The Hong Kong Polytechnic University, Hung Hom, Kowloon, Hong Kong 999077, China; lei2021.yuan@connect.polyu.hk (L.Y.); cyqni@polyu.edu.hk (Y.N.); 3 Institute of Urban Smart Transportation & Safety Maintenance, College of Civil and Transportation Engineering, Shenzhen University, Shenzhen 518060, China

**Keywords:** diffuse ultrasonic waves, fiber Bragg grating, damage detection, high-speed railway

## Abstract

Damage detection of railway tracks is vital to ensure normal operation and safety of the rail transit system. Piezoelectric sensors, which are widely utilized to receive ultrasonic wave, may be disturbed in the railway system due to strong electromagnetic interference (EMI). In this work, a hybrid ultrasonic sensing system is proposed and validated by utilizing a lead-zirconate-titanate (PZT) actuator and a fiber Bragg grating (FBG) sensor to evaluate damage conditions of the railway tracks. The conventional ultrasonic guided wave-based method utilizing direct wave to detect damages is limited by the complex data analysis procedure and low sensitivity to incipient damage. Diffuse ultrasonic wave (DUW), referring to later arrival wave packets, is chosen in this study to evaluate structural conditions of railway tracks due to its high sensitivity, wider sensing range, and easy implementation. Damages with different sizes and locations are introduced on the railway track to validate the sensitivity and sensing range of the proposed method. Two damage indices are defined from the perspective of energy attenuation and waveform distortion. The experimental results demonstrate that the DUW signals received by the hybrid sensing system could be used for damage detection of the railway tracks and the waveform-distortion-based index is more efficient than the energy-based index.

## 1. Introduction

**Figure 1:**
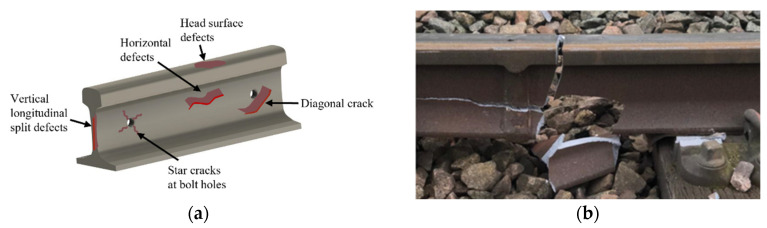
(**a**) Schematic and (**b**) in situ illustration of rail track damages.

Rail transit has developed dramatically worldwide due to its convenience for people’s daily lives. However, railway tracks are fragile in regard to defects because of high-speed operation, heavy loads, environmental exposure, and unpredictable impacts. Typical defects of railway tracks are shown in Figure 1. Catastrophic accidents may occur if defects cannot be detected [1,2].

Many nondestructive testing (NDT) techniques [3,4,5,6] have been explored and applied in the daily inspection of railway tracks combined with manual inspection. Among them, the ultrasonic bulk wave method with devices installed on the track inspection vehicle is well commercialized in routine inspection and maintenance for railway tracks [7]. Nevertheless, implementation of regular NDT techniques needs to interrupt the normal operation of the railway system, which is inconvenient, time consuming, and insecure for the inspectors. Furthermore, NDT techniques cannot monitor the conditions of the railway tracks in real time and provide timely alarms.

To improve the drawbacks of NDT techniques, acoustic emission (AE) and ultrasonic guided wave-based methods have attracted more attention in past years. AE has shown its effectiveness in railway crack monitoring [8,9]. However, AE signals suffer a low signal-noise ratio (SNR) and are insensitive to cracks that expand at a low rate. Although artificial intelligence techniques have been applied to AE wave classification and mass data processing [10,11], AE techniques still face the problem of ambient noise.

Ultrasonic guided wave method adopts an active manner to monitor structural conditions, which makes this method immune to most noise in condition monitoring of the railway system [12,13,14,15,16]. The ultrasonic guided wave is excited at a well-selected frequency and interacts with the defects. The wave reflection, transmission, mode conversion, and energy loss can be used for damage detection. However, multimode and dispersive features of ultrasonic guided wave in railway tracks make it difficult to extract damage information from recorded signals [17]. In addition, this method utilizes direct wave (first arriving wave packets) for damage detection, which leads to low sensitivity to incipient damage and limited sensing range. All the above factors impede wider application of this method to condition monitoring of railway tracks.

Different from the ultrasonic guided wave method, the diffuse ultrasonic wave (DUW)-based method utilizes later arrived wave packets (diffuse/coda wave) to monitor structural conditions. DUW has been neglected in past research due to its noise-like appearance. However, it was recently found that DUW is highly repeatable and carries more information about the medium [18,19]. DUW is very sensitive to small changes in the medium since it propagates for longer propagation distance and interacts with scattering sources (defects) multiple times. Compared to the direct wave, the DUW received by the sensor is the superposition of waves from all directions, which leads to the wider sensing range [20,21]; on the other hand, multiple scattering events make the DUW sensitive to small perturbations of the materials [22,23].

DUW was first explored in geological engineering to identify slight changes of the earth’s crust by seismologists [24]. Recently, efforts have been made to apply DUW to damage detection and condition monitoring of concrete materials [25,26,27], composite structures [23,28,29], and metallic structures [30,31]. Liu et al. [25] utilized DUW for self-healing process monitoring of concrete where biomineralization was used to repair internal cracks. The results indicated that the relative velocity change of the DUW could reveal the strength development of the self-healing concrete. Lim et al. [23] applied DUW to early-stage fatigue damage detection and crack growth monitoring of carbon-fiber-reinforced polymer (CFRP) composite plate. The results showed that time domain distortion of DUW signals could be used to assess fatigue damage of the CFRP plate. Ahn et al. [26] utilized DUW to evaluate distributed cracks in concrete. The results demonstrated that both diffusivity and dissipation coefficients of UGW signals could be used for micro cracks detection. The feasibility of DUW for damage detection has also been demonstrated on woven fabric composite structures [28] and aeronautical honeycomb composite sandwich structures [29]. In terms of metal structures, Xie et al. [30,31] proposed a DUW-based method to monitor temperature variations and thermal-shock-induced microstructural alterations in steel specimens.

It can be seen from the above research that DUW has been widely studied on condition monitoring, such as distributed cracks and microstructural changes of the medium. The corresponding results proved that DUW has good performance in quantifying these parameter changes. However, miniature local damage is more commonly seen and is essential for infrastructure. Recently, local damage detection using DUW has attracted more attention. Pacheco and Snieder [32] developed the DUW technique for local damage evaluation, but this method may not be adequate for small localized damages [33]. Fröjd and Ulriksen [21] utilized decorrelation of DUW signals in a specific time window to evaluate local holes in a concrete floor slab. However, the decorrelation coefficient calculation using a specific time window may not be as robust as using longer segments of recorded signal [34]. Michaels et al. [35] proposed local temporal coherence (*LTC*) to detect local damage in aluminum plate. It was found that *LTC* had good performance in small defect detection. Fröjd and Ulriksen [36] combined amplitude and phase information by establishing the Mahalanobis model to evaluate damages induced by impact hits on concrete slabs.

The studies above have shown the sensitivity of DUW to miniature and local changes in concrete and composite structures. However, few studies have explored the application of DUW to damage detection in railway tracks. Wang et al. [37,38] innovatively applied DUW to condition monitoring of railway turnouts. Remnant cross-correlation coefficients of the DUW signals were extracted to detect defects. This pioneering work provides a benchmark-free method for condition monitoring of railway turnouts. Nevertheless, the sensitivity to local damage and wide-range sensing capacity of DUW on railway tracks are not fully studied in this research.

Lead-zirconate-titanate (PZT) sensor is adequate for ultrasonic wave detection under normal conditions [39]. However, the railway system usually has strong electromagnetic interference (EMI), which might reduce the SNR of ultrasonic signals received by the PZT sensor. On the other hand, the connection wires of the PZT sensor for voltage delivery limit its sensing range and multipoint installation for distributed sensing. Furthermore, the dielectric constant and silver cladding of the PZT sensors will be easily degraded under long-term environmental exposure [40]. These factors impede the wider application of PZT sensor on ultrasonic sensing of railway tracks. Recently, fiber Bragg grating (FBG) sensors have been explored to receive ultrasonic waves due to their advantages of being lightweight, having the potential to multiplex and be immune to EMI, moisture, and high temperatures. Moreover, the FBG sensor is applicable for locations with complex shapes where PZT transducers are hard to access. Tian et al. [41] established FBG array to receive Lamb wave and visualize damages on aluminum plate. Wang and Wu [42,43] applied phase-shift FBG sensor to obtain ultrasonic signals, and the nonlinearity of ultrasound was utilized to evaluate fatigue cracks. Yu et al. [44] took advantage of the high-temperature resistance of FBG sensors to detect damage at very high temperature. The research above proved the FBG sensor’s immunity to harsh environments and high sensitivity to ultrasonic wave. Cano et al. [45] successfully verified the feasibility of FBG sensors for receiving ultrasonic waves on subway rail specimens. Wang et al. [46] explored the optimal excitation frequency of ultrasonic guided waves for damage detection on rails by using FBG sensors. However, the application of FBG sensors for DUW-based damage detection on railway tracks has not yet been investigated.

In this paper, a hybrid sensing system with PZT actuator and FBG sensor is proposed to obtain DUW on railway tracks for damage detection. Laboratory tests are conducted on a segment of a 60 kg/m railway track to investigate the sensitivity and sensing range of DUW. Damage indices based on energy attenuation and waveform distortion of DUW signals are proposed and validated to quantify different damage levels. This work will contribute a new sensing system for damage detection of railway tracks and provide a deep understanding of the interaction between damage and DUW.

## 2. Methodology

**Figure 2:**
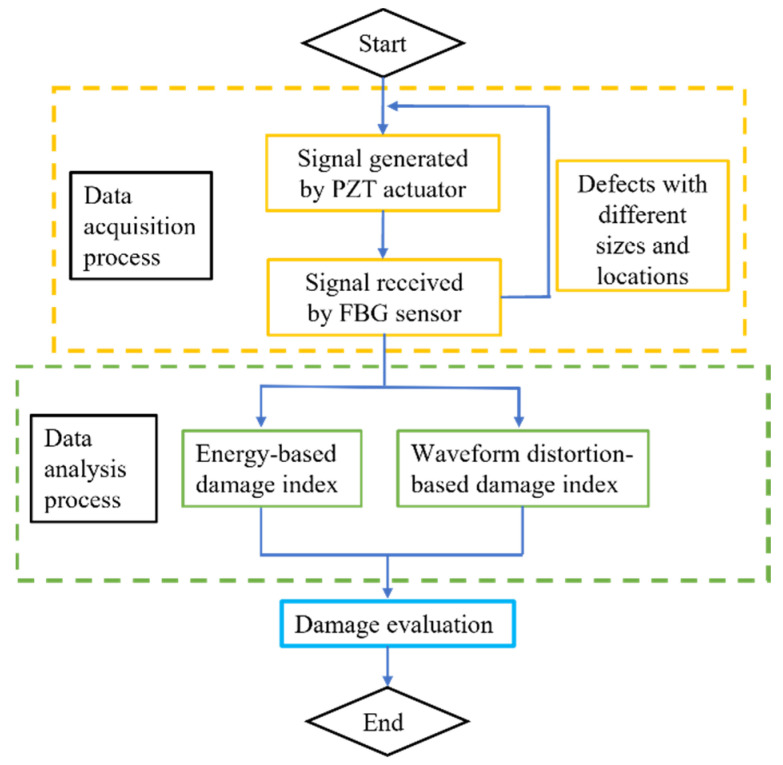
Flowchart of the proposed damage evaluation method on railway tracks.

DUW is chosen to monitor the conditions of railway tracks based on the PZT/FBG hybrid sensing system. Different damage levels are introduced by attaching Blu Tack blocks on the track web to explore the sensitivity and sensing range of this method. Defects will cause not only energy attenuation but also waveform distortion of the DUW signal. Therefore, energy-based and waveform distortion-based damage indices are respectively defined to indicate conditions of the railway track. A flowchart of the proposed method is presented in Figure 2.

### 2.1. Working Principle of FBG Sensor

**Figure 3:**
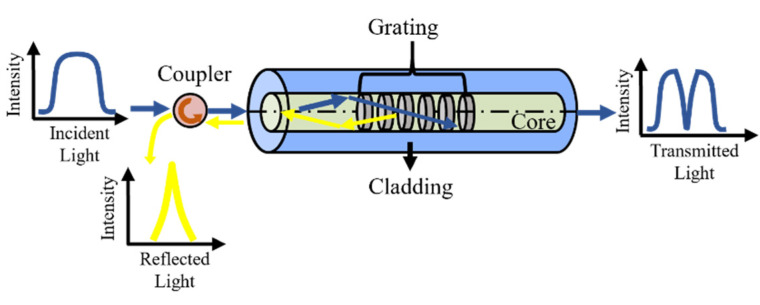
Principle of FBG sensor.

Fiber Bragg grating (FBG) is a fiber optic sensor that has periodic changes in the refractive index of the fiber core. The periodical grating structures could act as a narrowband filter, and the wavelength of reflected light is called the Bragg wavelength, which can be expressed by:

(1) $$\lambda_{b}=2n\Lambda$$

where *λ_b_* is the Bragg wavelength of FBG, *n* is the effective refractive index of the optical fiber, and Λ is the grating period. When broadband light is propagated into the FBG sensor, light with central wavelength *λ_b_* will be reflected, while other components will be transmitted through the grating, as shown in Figure 3.

**Figure 4:**
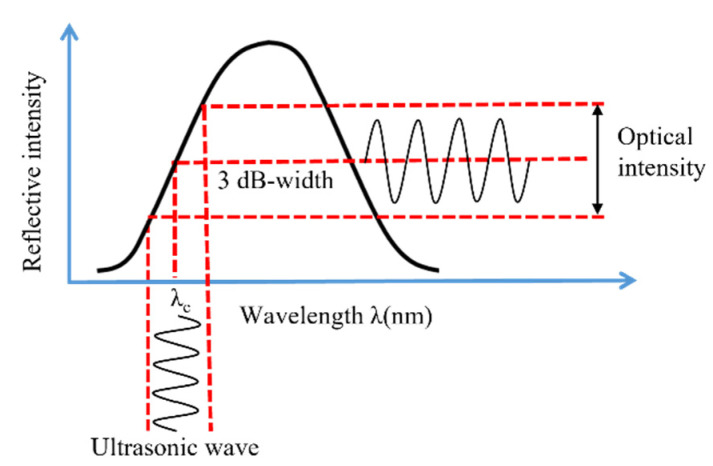
FBG demodulation techniques using a narrowband light source.

Micro vibration induced by the ultrasonic wave will cause a Bragg wavelength shift and the relationship between the Bragg wavelength shift and strain along the fiber direction without temperature variation can be represented by [47]:

(2) $$\Delta \lambda_{b}/\lambda_{b}=C_{\varepsilon }\varepsilon_{z}$$

where Δ*λ_b_* is the Bragg wavelength shift, *C_ε_* is the material constant obtained from calibration experiments, and *ε_z_* is the strain along the fiber axis. The conventional optical spectrum analyzer is unqualified to capture high-frequency vibration induced by ultrasonic wave due to the limitation of the demodulation speed. The two most prevailing FBG demodulation techniques for ultrasonic detection are intensity demodulation technique and edge filter demodulation technique [48]. The light source for intensity demodulation is broadband light source, while for edge filter demodulation, it is narrowband light source. Even though intensity demodulation has potential application for multiplexing, its SNR is relatively low. On the other hand, the edge filter technique has been widely used in ultrasonic detection due to the high signal quality [49,50,51]. The demodulation principle of edge filter technique is adopted in this study and can be explained in Figure 4. The light source wavelength is locked at the 3 dB point of the FBG spectrum. The intensity of the reflected light will change with the Bragg wavelength shift induced by micro vibration and be proportional to the amplitude of the ultrasonic wave.

It has been proven that the dominant noise of the FBG ultrasonic sensing system is laser intensity noise [44]. To reduce this noise and improve the SNR, a balanced photodetector (BPD) is utilized to receive both reflection and transmission signals of the FBG. The voltage obtained by two parts of BPD would simultaneously experience changes with the same amplitudes but opposite phases [52]. Therefore, BPD could double the amplitude of the signal while removing noise when both transmitting and reflecting light pass into the connectors. The voltage obtained by BPD could be given by:

(3) $$V=2\Delta \lambda_{b}GR_{D}Pg$$

where *V* is the output voltage of the BPD, *G* is the grating slope, *R_D_* and *g* are the response and gain factor of the BPD, respectively, and *P* is the laser power of the tunable laser resource.

### 2.2. Principle of DUW on Condition Monitoring of Railway Track

#### 2.2.1. DUW Propagation in Railway Track

Many studies have utilized direct wave to obtain clear responses by generating and extracting pure mode of ultrasonic guided waves [53,54]. However, methods for exciting the ideal mode and minimizing dispersion in railway tracks need to be further studied [55].

**Figure 5:**
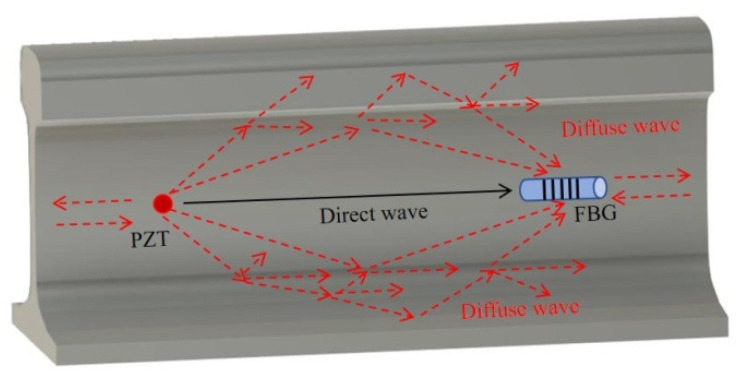
Schematic illustration of ultrasonic wave field on rail.

Different from the direct wave, the DUW-based method utilizes later wave packets that are reflected and scattered multiple times. A schematic illustration of the diffuse ultrasonic wave field on a railway track is shown in Figure 5. The low acoustic attenuation coefficient of steel allows ultrasonic wave to propagate for a longer time. The DUW method utilizes the change of diffused ultrasonic wave field that consists of many modes for damage detection.

#### 2.2.2. Energy-Based Damage Index of Diffuse Wave

The energy-based damage index has been widely used in ultrasound-based damage detection [40,56]. As discussed above, local damage in the medium will lead to energy attenuation of DUW. In this research, a damage index derived from wavelet-packet-based energy (WPE) analysis [57,58] is proposed to evaluate the damage severity of railway tracks. In general, the DUW signal *X* will be decomposed into $2^{n}$ frequency bands by *n*-level wavelet packet decomposition. The *j*-th frequency band *X_j_* could be given by:

(4) $$X_{j}=[X_{j,1},X_{j,2},\ldots ,X_{j,m}]$$

where *j* represents frequency bands varying from 1 to $2^{n}$, and *m* is the total amount of data. Then, the wavelet packet energy of the *j*-th frequency band *E_j_* can be calculated by:

(5) $$E_{j}=X_{j,1}^{2}+X_{j,2}^{2}+\ldots +X_{j,m}^{2}$$

The energy vector *E* of the DUW is given by:

(6) $$E=[E_{1},E_{2},\ldots ,E_{2^{n}}]$$

Then, the energy-based damage index (*EDI*) based on WPE can be further defined by:

(7) $$EDI=\sqrt{\sum_{j=1}^{2^{n}}{\left(E_{j}-E_{intact,j}\right)}^{2}/\sum_{j=1}^{2^{n}}E_{intact,j}{}^{2}}$$

where *E_intact,j_* is the energy of the *j*-th frequency band. If no change occurs in the medium, *E_intact,j_* is close to *E_j_*, and the *EDI* will approach 0. If a strong scattering source exists in the medium, *E_j_* will be strongly different from *E_intact,j_*, and the *EDI* will approach 1.

#### 2.2.3. Waveform-Distortion-Based Damage Index of Diffuse Wave

DUW has been widely used in global damage detection by using time delay or relative velocity change to quantify the global compressing or stretching of DUW waveforms. However, those studies are established on the assumption that changes in the medium are global, which is inadequate for local damage detection.

**Figure 6:**
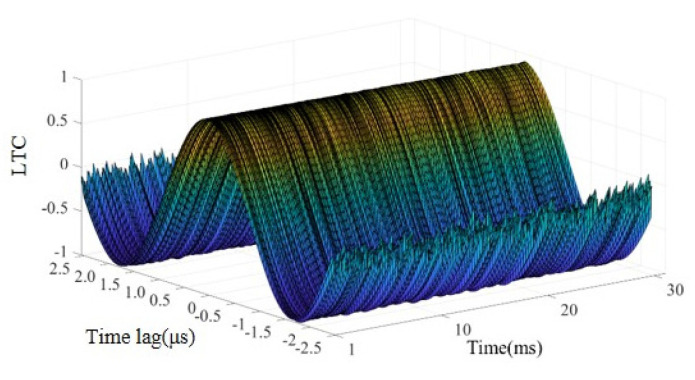
Local temporal coherence (*LTC)* of example signal.

Local temporal coherence (*LTC*), which has shown its effectiveness in quantitatively describing signal changes induced by local damage [35], is defined as:

(8) $$LTC^{\left(T_{0}\right)}(\tau )=\frac{\int_{T_{0}-T}^{T_{0}+T}X_{1}(t)\cdot X_{2}(t+\tau )dt}{\sqrt{{\int_{T_{0}-T}^{T_{0}+T}X_{1}(t)}^{2}dt\cdot \int_{t1}^{t2}X_{2}{\left(t+\tau \right)}^{2}dt}}$$

where *X*_1_(*t*) is the baseline signal and *X*_2_(*t*) is the signal obtained from the damaged condition. *LTC* values quantify the correlation between two signals in a time window. The damaged signal is first translated by *τ* in time domain, and similarity of the two signals is calculated in time window [T_0_ − T, T_0_ + T]. *τ* values ranging from −2.5 μs to 2.5 μs in step of 0.005 μs are used to calculate *LTC* in this study. The length of the time window is set to 0.4 ms, which is 10 times the excitation signal length. The time window is moved along the time axis in step of 0.05 ms to obtain the *LTC* values of the entire signal. An example envelope of LTC between the baseline signal and measured signal is shown in Figure 6. It is noted that waveforms in the first 1 ms are discarded since the ultrasonic wave at the very beginning has low sensitivity to damage.

Peak coherence (*PC*) represents the maximum *LTC* value with respect to τ at each time window, and it can be given by

(9) $$PC(t)=\underset{\tau }{\max}(LTC(\tau ,t))$$

**Figure 7:**
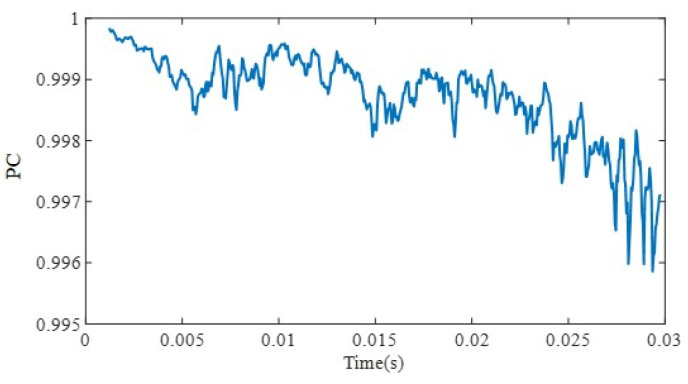
Peak coherence of example signal.

*PC* values calculated from signals in Figure 6 are shown in Figure 7.

Aiming at quantitatively describing the distortion degree of the signals, peak coherence change (*PCC*) is defined as the difference between the maximum *PC* value and the average *PC* value, and it can be given by:

(10) PCC=max[PC(t)]−mean[PC(t)]

It is noted that temperature effect could be discriminated in the process of calculating the *PCC* value [59]. Therefore, temperature variation could be removed, and only defect information would remain in the *PCC* values.

## 3. Experiment Procedure

To verify the feasibility of applying DUW to damage detection of railway tracks using the PZT/FBG hybrid sensing system and demonstrate its sensing range and sensitivity, a series of laboratory tests is conducted on a section of a 60 kg/m railway track with a 400 mm length.

### 3.1. PZT/FBG Hybrid Ultrasonic Sensing System

**Figure 8:**
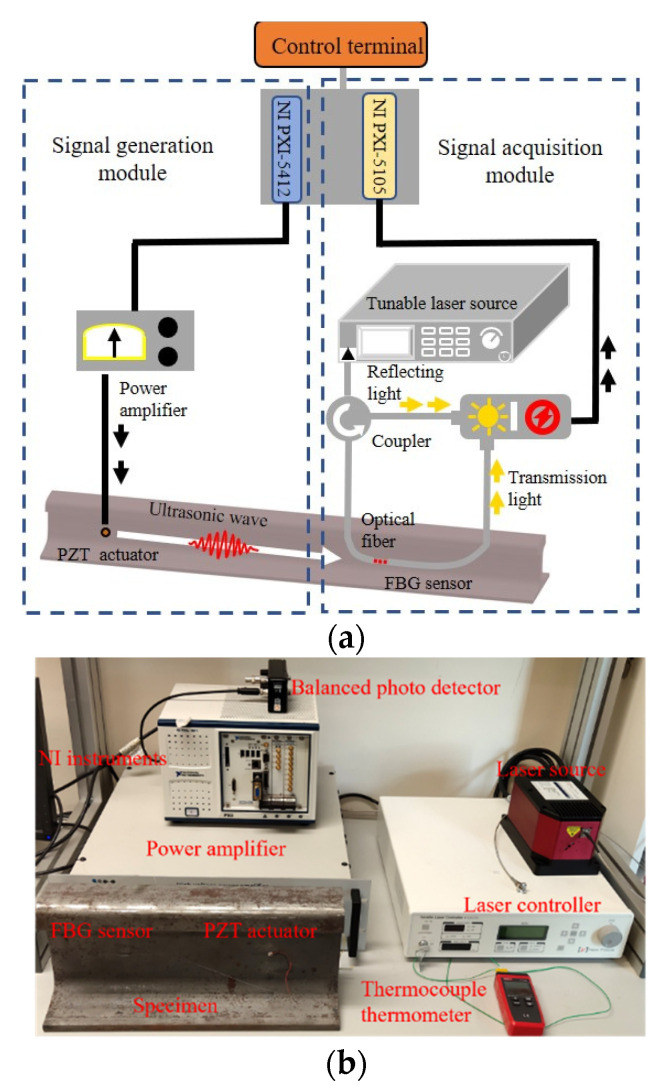
Experimental setup of the PZT/FBG hybrid sensing system: (**a**) schematic illustration of the experimental setup; (**b**) laboratory setup.

The proposed sensing system in this study consists of the signal generation module and the signal acquisition module, as shown in Figure 8. The ultrasonic wave is generated by the typical PZT-based ultrasonic generating system, and the ultrasonic wave will propagate along the railway track. The micro vibration induced by the ultrasonic wave will be perceived by the FBG sensor, and the structural condition can be evaluated by analyzing the response signal.

**Figure 9:**
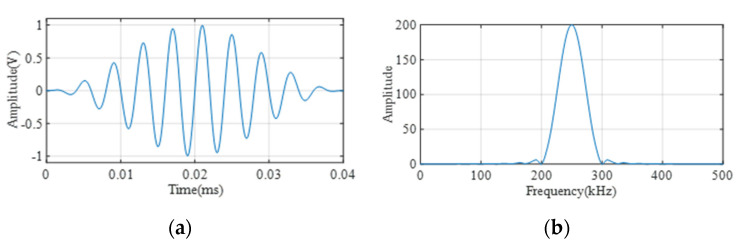
Excitation ultrasonic signal in: (**a**) time domain; (**b**) frequency domain.

The detailed working procedure of the whole PZT/FBG hybrid ultrasonic sensing system is as follows: A 250 kHz, ten-cycle sinusoidal tone burst modulated by a Hanning window is first generated by an arbitrary waveform generator (PXI-5412, National Instruments, Austin, TX, USA), as shown in Figure 9. The excitation signal is amplified 200 times using a linear power amplifier (HVA-400-A, Ciprian, La Tronche, France). The amplified signal is sent to the PZT disc (diameter: 8 mm, thickness: 1 mm) to generate ultrasonic waves. An optical spectrum analyzer (AQ6370D, Yokogawa, Tokyo, Japan) is used to obtain the reflecting spectrum of FBG, and a tunable laser (TLB-6700, Newport, RI, USA) is utilized to emit a narrowband light source according to the FBG reflecting spectrum. In this study, the laser wavelength is set at 1556.07 nm, which is the 3 dB position on the left-hand side of the FBG spectrum. The micro strain induced by ultrasonic waves will shift the Bragg wavelength and cause changes in the optical intensity. Both the transmitted and reflected light of the FBG sensor are guided into two parts of the BPD (2117-FC, Newport, RI, USA), which converts the optical signal into a voltage signal. The voltage signals are obtained by the oscilloscope (PXI-5412, National Instruments, Austin, TX, USA) and the sampling frequency of the oscilloscope is set at 20 MHz.

Considering that the frequency of the excitation signal is 250 kHz, the wavelength of the ultrasonic wave with different modes ranges from approximately 10 mm to 20 mm, and an FBG sensor with a 10 mm grating length is adopted in this study. The whole procedure is controlled by LABVIEW software, and data analysis is conducted in MATLAB software.

### 3.2. Experiment Setup

**Figure 10:**
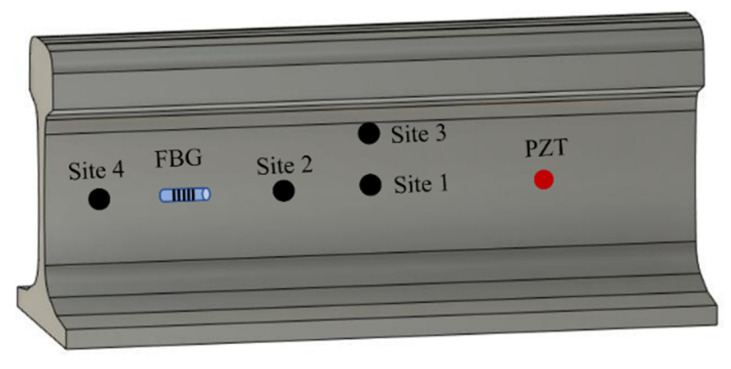
Schematic illustration of the testing specimen and damage location.

Both PZT actuator and FBG sensor are attached on the rail web using epoxy adhesive, and the distance between the PZT and FBG is 200 mm. Blu Tack blocks are utilized to introduce damages in the rail web, which has demonstrated effectiveness in simulating defects in many studies [60,61]. The diameters of defects are set as 2.5 mm, 5 mm, 7.5 mm, 10 mm, 20 mm, and 30 mm. Defects smaller than 10 mm are considered sub-wavelength defects, while defects larger than 10 mm are regarded as over-wavelength defects. Damages are set at four different locations on the rail web. As shown in Figure 10, site 1 and site 2 are located at the direct sensing path between the PZT actuator and FBG sensor. Sites 3 and 4 are placed outside the direct sensing path.

The intact railway track is first tested to obtain the baseline signals. Measurements are then conducted on each damaged condition. A thermocouple is placed on the rail surface to measure the temperature, and an air conditioner is used to ensure that the room temperature is constant for all tests. It needs to be noted that the thermal variations in practice will shift the FBG peak, which may affect the reflective intensity of signals. To eliminate the temperature effect, a preliminary test can be conducted to obtain the central wavelength of FBG before each ultrasonic measurement. The 3 dB point of the FBG spectrum can then be determined accurately for adjusting the laser wavelength.

## 4. Experimental Results and Discussion

Signals with a length of 30 ms are recorded in each measurement. Each condition is measured ten times, and the signals are filtered and smoothed using a Butterworth band-pass filter through a toolbox in MATLAB. The filtered signals in each condition are further averaged to reduce stochastic noise.

**Figure 11:**
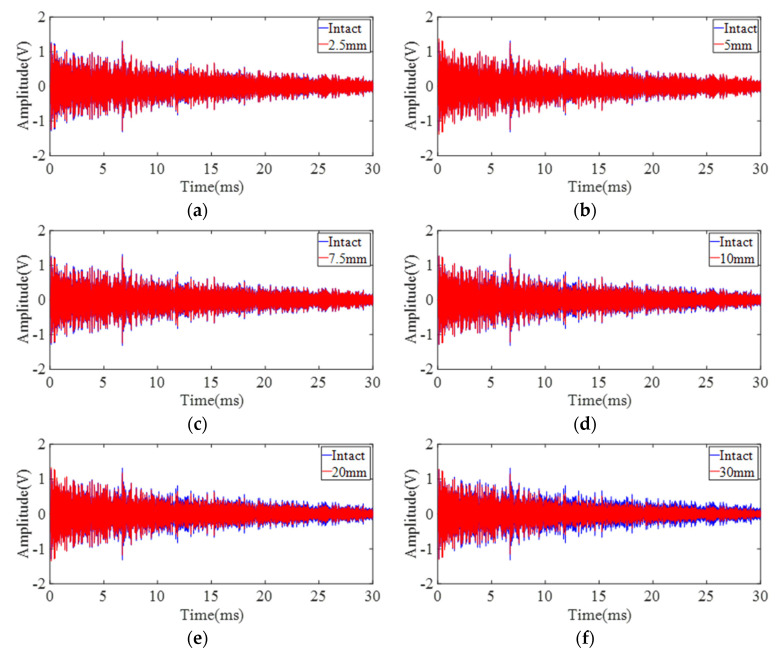
Signals obtained from six configurations on the middle point of the direct sensing path (site 1): (**a**) 2.5 mm defect; (**b**) 5 mm defect; (**c**) 7.5 mm defect; (**d**) 10 mm defect; (**e**) 20 mm defect; (**f**) 30 mm defect.

**Figure 12:**
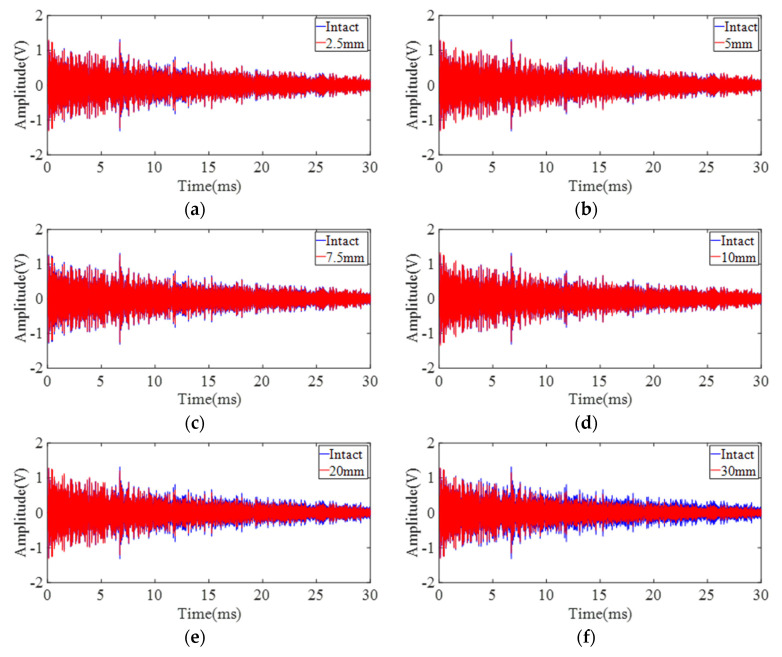
Signals obtained from six configurations on the left point of the direct sensing path (site 2): (**a**) 2.5 mm defect; (**b**) 5 mm defect; (**c**) 7.5 mm defect; (**d**) 10 mm defect; (**e**) 20 mm defect; (**f**) 30 mm defect.

**Figure 13:**
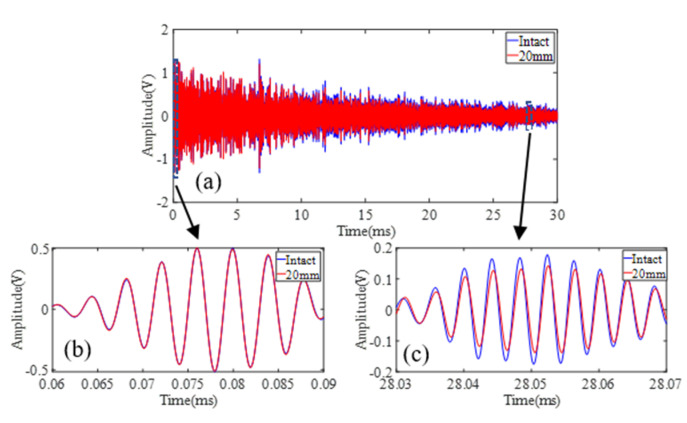
Signals obtained from intact rail (blue) and 20 mm condition (red): (**a**) full-length signal; (**b**) direct wave; (**c**) diffuse ultrasonic wave.

Experiments are first conducted on site 1 and site 2 to demonstrate the feasibility of the proposed sensing system when damage is located along the direct sensing path. DUW obtained from site 1 and site 2 is shown in Figure 11 and Figure 12. In general, the differences between the disturbed signals and baseline signals become larger with increasing damage size at both site 1 and site 2. A detailed presentation of the signals in Figure 12e is shown in Figure 13. It is obvious that direct wave (Figure 13b) is almost identical while DUW signals (Figure 13c) vary in both amplitude and phase. The comparison between direct wave and DUW shows the high sensitivity of DUW for damage detection.

For damage quantification, the energy-based damage index (*EDI*) is obtained for different configurations, as shown in Equation (7). Since the differences between the measured and baseline signals increase with time, which demonstrates that damage information accumulates in the wave propagation process, different segments of signals to be analyzed may lead to different results. Therefore, three configurations of time window, including 1–10 ms, 10–20 ms, and 20–30 ms, are utilized to calculate the *EDI* values.

**Figure 14:**
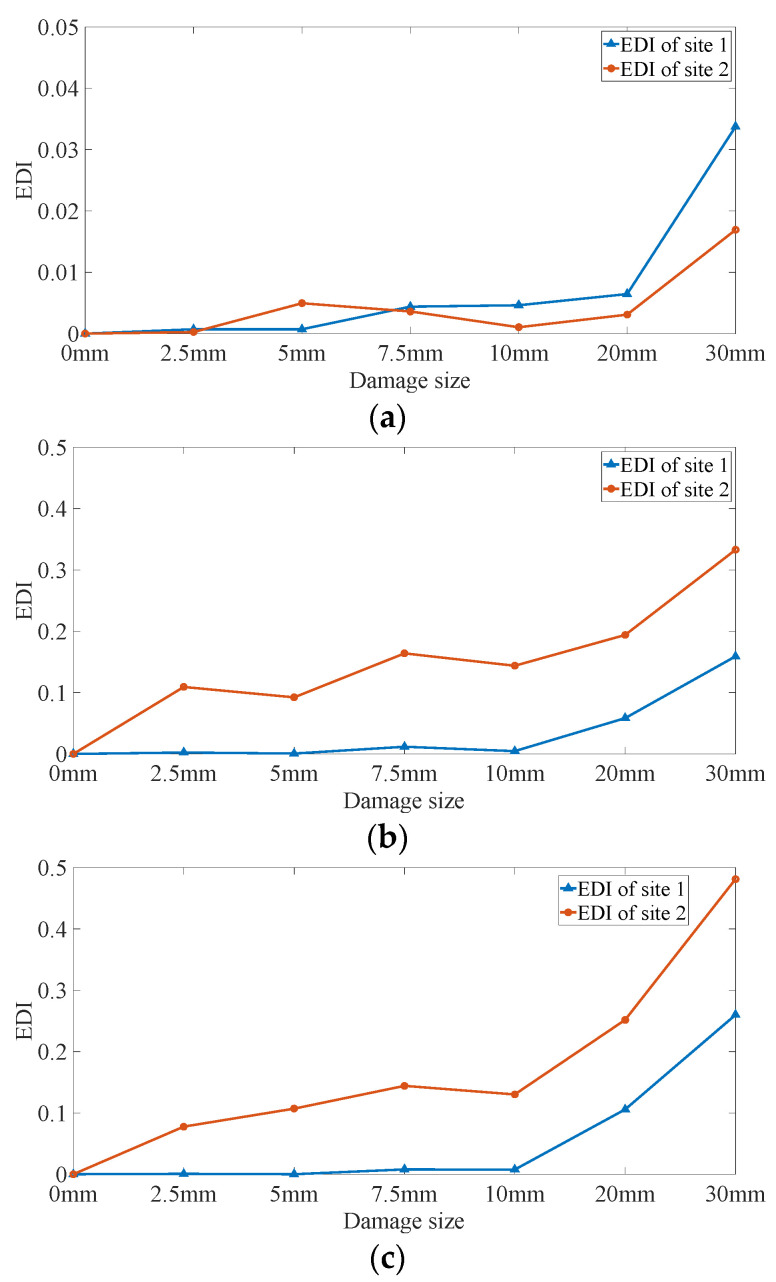
Energy-based damage index of DUW signals: (**a**) 1–10 ms, (**b**) 10–20 ms, and (**c**) 20–30 ms.

As shown in Figure 14, the calculated *EDI* values do not monotonically increase with damage size. Specifically, *EDI* values fluctuate when the defect sizes are smaller than 10 mm and increase significantly in 20 mm and 30 mm conditions in all the three time-window configurations. The reason is that over-wavelength defects cause more significant energy attenuation than sub-wavelength ones, and *EDI* could reflect the global features rather than the detailed information of the signals, which makes it adequate for severe damage detection but inefficient in quantifying the incipient damage.

**Figure 15:**
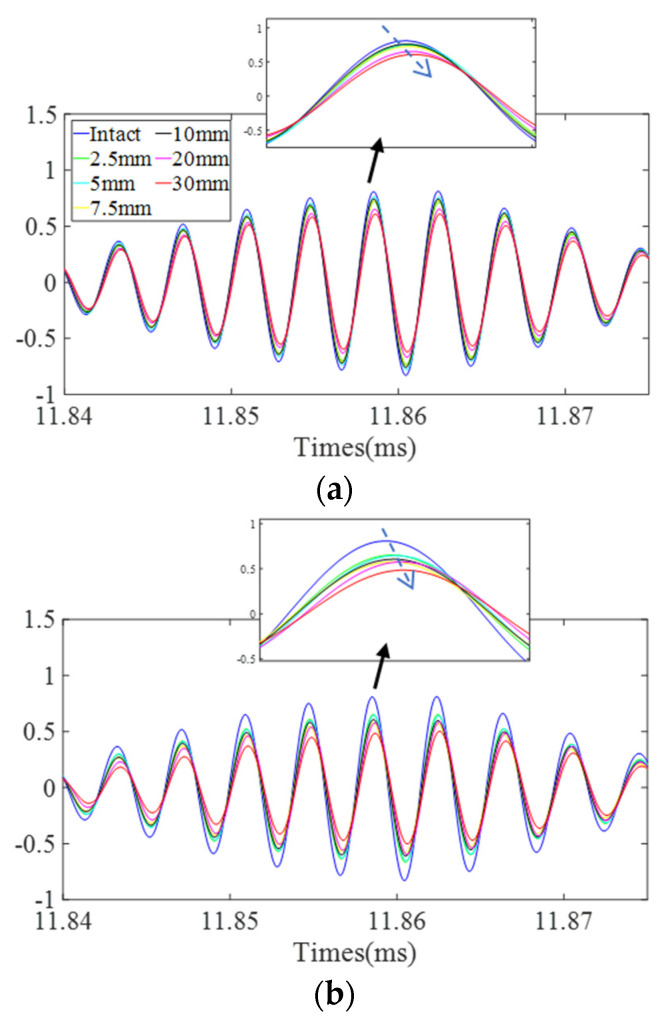
Variation of ultrasonic wave: (**a**) site 1; (**b**) site 2.

Figure 15 shows the variations of DUW signals in a specific time window of site 1 and site 2 with different damage sizes. It is obvious that waveforms in this time window are locally distorted rather than globally stretched or compressed. On the other hand, defects with different sizes will distort the waveform to different degrees. The signals in the time domain show that railway track damage might be quantitatively described by evaluating the waveform distortion degrees of DUW signals.

**Figure 16:**
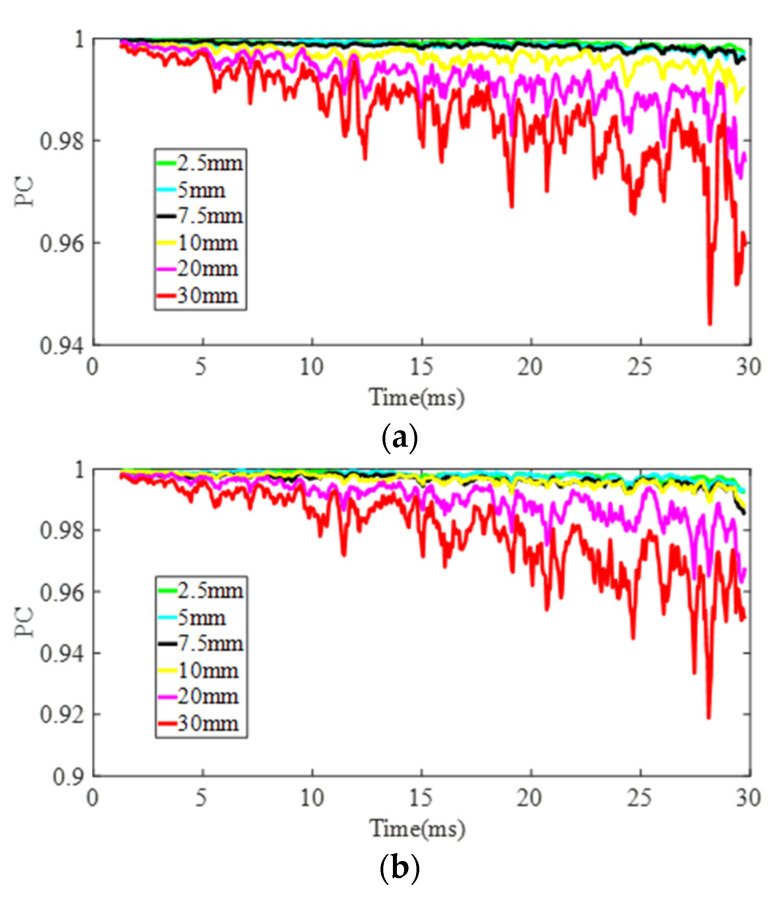
Peak coherence in each condition: (**a**) damage is located at the middle point of the direct sensing path and (**b**) damage is located at the left point of the direct sensing path.

The *LTC* values, which are obtained from the coherence of the measured signal and the baseline signal, are then extracted under every condition using the method proposed in Section 2.2.3. *PC* and *PCC* values of signals are calculated based on *LTC* values to quantitatively present variations of DUW signals under different damage sizes. As shown in Figure 16, *PC* values drop as a function of time, which meets well with the results obtained by Michaels [36] and Lu [60] and indicates that defect information accumulates in the process of DUW propagation. Moreover, *PC* values decrease with increasing defect size at both site 1 and site 2.

**Figure 17:**
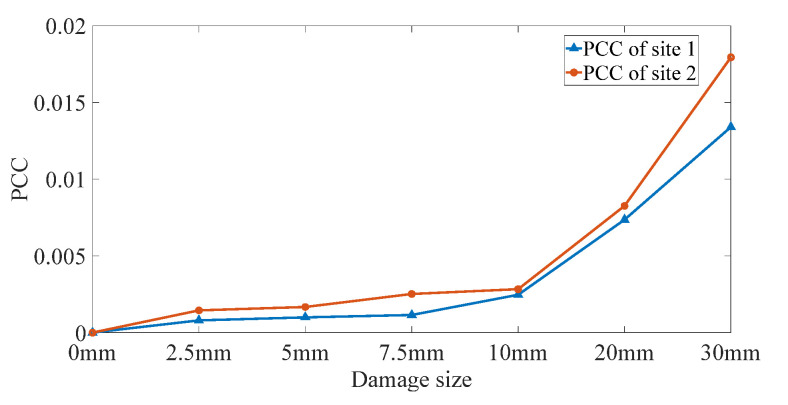
Damage index based on *PCC* of site 1 and site 2.

The *PCC* values are then calculated from *PC* results to quantify the damages in site 1 and site 2, and the results are shown in Figure 17. In general, *PCC* values increase with the damage size in all the damage conditions, which proves that *PCC* is more effective than *EDI* in damage quantification. Specifically, *PCC* develops slowly when the defect diameter is smaller than 10 mm. On the one hand, the increasing speed of damage from 2.5 mm to 10 mm is relatively low, which causes the slow development of *PCC* values; on the other hand, multiple interactions between the waves and defects cause the subtle changes to be perceived by DUW. The *PCC* increases dramatically when the damage sizes are larger than 10 mm. The reason is that defects will strongly interact with DUW when the sizes are close to the wavelength of ultrasonic waves. The results in Figure 17 demonstrate that the damages located at the direct sensing path could be detected by using the proposed method.

**Figure 18:**
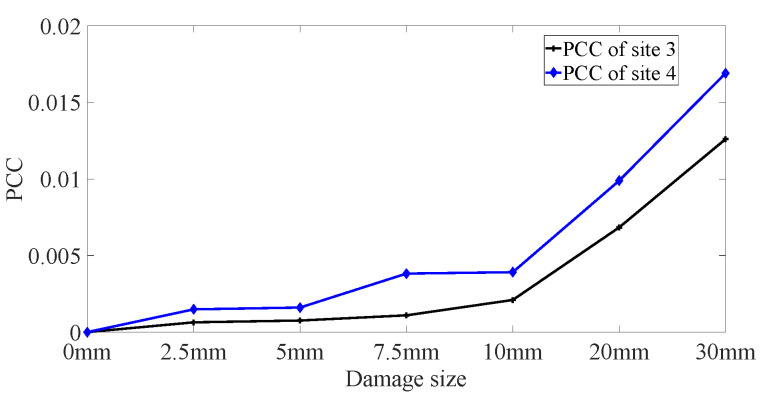
Damage index based on *PCC* of site 3 and site 4.

DUW propagates through not only the direct path between PZT and FBG but also the paths outside the direct path, which results in DUW being able to detect damage beyond the direct path. Therefore, the *PCC* values of signals obtained from site 3 and site 4 are obtained to investigate the sensing range of DUW, as shown in Figure 18. *PCC* values increase with the damage size in both site 3 and site 4, which verifies the wide sensing capacity of DUW.

## 5. Conclusions

This study explores DUW for damage detection of railway tracks using a PZT/FBG hybrid sensing system. The sensitivity and sensing range of the proposed method are investigated by a series of experiments. The conclusions of this research can be summarized as follows:

(1) The PZT/FBG hybrid sensing system is adequate for damage detection in railway tracks. The sensitivity of direct wave and DUW is compared in the time domain. Variations of DUW signals are much larger than direct wave signals, which demonstrates the higher sensitivity of DUW than direct wave for damage detection.
(2) The energy-based damage index, *EDI*, is first defined and utilized to quantify the damage severity at sites 1 and 2. *EDI* is sufficient to evaluate over-wavelength defects but is not adequate for sub-wavelength defects.
(3) The waveform-distortion-based damage index, *PCC*, is defined and utilized for damage detection on railway tracks. The results show that the *PCC* values increase with damage size at all four sites, and the damage index is efficient for damage detection.

In the future, environmental effects such as temperature and moisture on the proposed system need to be studied. Advanced signal processing techniques should be considered to make full use of the abundant information in diffuse ultrasonic signals. Longer railway tracks also need to be studied to explore the sensing range along the direction length of railway tracks of the proposed method.
